# The Influence of Kinematic Constraints on Model Performance During Inverse Kinematics Analysis of the Thoracolumbar Spine

**DOI:** 10.3389/fbioe.2021.688041

**Published:** 2021-07-29

**Authors:** Mohammad Mehdi Alemi, Katelyn A. Burkhart, Andrew C. Lynch, Brett T. Allaire, Seyed Javad Mousavi, Chaofei Zhang, Mary L. Bouxsein, Dennis E. Anderson

**Affiliations:** ^1^Center for Advanced Orthopaedic Studies, Beth Israel Deaconess Medical Center, Boston, MA, United States; ^2^Department of Orthopaedic Surgery, Harvard Medical School, Boston, MA, United States; ^3^Department of Automotive Engineering, Tsinghua University, Beijing, China

**Keywords:** spine motion, degrees of freedom, dynamic movement, smoothness of motion, optoelectronic motion capture

## Abstract

Motion analysis is increasingly applied to spine musculoskeletal models using kinematic constraints to estimate individual intervertebral joint movements, which cannot be directly measured from the skin surface markers. Traditionally, kinematic constraints have allowed a single spinal degree of freedom (DOF) in each direction, and there has been little examination of how different kinematic constraints affect evaluations of spine motion. Thus, the objective of this study was to evaluate the performance of different kinematic constraints for inverse kinematics analysis. We collected motion analysis marker data in seven healthy participants (4F, 3M, aged 27–67) during flexion–extension, lateral bending, and axial rotation tasks. Inverse kinematics analyses were performed on subject-specific models with 17 thoracolumbar joints allowing 51 rotational DOF (51DOF) and corresponding models including seven sets of kinematic constraints that limited spine motion from 3 to 9DOF. Outcomes included: (1) root mean square (RMS) error of spine markers (measured vs. model); (2) lag-one autocorrelation coefficients to assess smoothness of angular motions; (3) maximum range of motion (ROM) of intervertebral joints in three directions of motion (FE, LB, AR) to assess whether they are physiologically reasonable; and (4) segmental spine angles in static ROM trials. We found that RMS error of spine markers was higher with constraints than without (*p* < 0.0001) but did not notably improve kinematic constraints above 6DOF. Compared to segmental angles calculated directly from spine markers, models with kinematic constraints had moderate to good intraclass correlation coefficients (ICCs) for flexion–extension and lateral bending, though weak to moderate ICCs for axial rotation. Adding more DOF to kinematic constraints did not improve performance in matching segmental angles. Kinematic constraints with 4–6DOF produced similar levels of smoothness across all tasks and generally improved smoothness compared to 9DOF or unconstrained (51DOF) models. Our results also revealed that the maximum joint ROMs predicted using 4–6DOF constraints were largely within physiologically acceptable ranges throughout the spine and in all directions of motions. We conclude that a kinematic constraint with 5DOF can produce smooth spine motions with physiologically reasonable joint ROMs and relatively low marker error.

## Introduction

Spinal disorders, particularly low back pain (LBP), are key global health problems in both workplace and clinical settings with devastating effects on functional independence and work capacity, leading to disability and high medical and societal costs. For instance, LBP accounts for ~40% of lost workdays with an estimated direct healthcare expenditure of $50–90 billion annually in the US (Guo et al., 1999; Yang et al., 2016). Many spinal disorders, including idiopathic back pain, degenerative disc disease, lumbar spinal stenosis, vertebral fractures (traumatic or osteoporotic), spine deformity, and muscle imbalance (e.g., myopathy, muscle dystrophy), can alter the kinematics and posture of the trunk (Al-Eisa et al., 2006; Briggs et al., 2007; Mahaudens et al., 2009; Galvis et al., 2016; Kuwahara et al., 2016; Schmid et al., 2016; Basques et al., 2017; Christe et al., 2017; Chun et al., 2017; Crawford et al., 2018; Igawa et al., 2018). Therefore, objective measurements of trunk kinematics and posture are useful in evaluating the functional impacts of spinal disorders and the development of novel clinical treatments. Typically, spine alignment and posture are studied as an overall trunk angle. However, direct measurement of individual vertebral movement is possible and can provide added information on spine biomechanics in health and disease. For instance, *in vivo* measurements of intervertebral motion can be obtained using various techniques such as biplane fluoroscopy (Lin et al., 2014; Wang et al., 2020), videofluoroscopy (Wong et al., 2006; Cheng et al., 2016), standard radiographs (Cheng et al., 2016), CT scans (Cheng et al., 2016), and MRI (Fujii et al., 2007). However, such methods are costly and highly invasive, making them impractical for widespread use for either clinical or research purposes.

Optoelectronic motion analysis is a standard technique to measure body kinematics and is often implemented in studies of upper and lower extremity motions. Several studies have already reported the use of optoelectronic motion capture systems (such as Vicon Nexus) for measurement of trunk posture and motion (Rast et al., 2016; Sung et al., 2016; Ignasiak et al., 2017; Marich et al., 2017; Zwambag et al., 2019). However, there is no standardized approach for measurement of trunk posture and spinal motion due to the methodological differences [e.g., different marker location, marker set (single or clusters), and the number of markers on the spine] involved in generating reproducible spinal kinematics (Mason et al., 2016). Some studies have addressed the between-session reliability of motion capture for trunk posture and range of motion (ROM) measurements (Dunk et al., 2004, 2005; O'Sullivan et al., 2010; Fortin et al., 2012; Hidalgo et al., 2012; Harsted et al., 2016; Rast et al., 2016; Muyor et al., 2017; Mousavi et al., 2018). Overall, these studies provide some evidence that optoelectronic motion capture data may provide an indirect but reliable approach to non-invasively assess the kinematics of the spine.

Although optoelectronic motion analysis systems allow overall measurement of trunk posture and motion, it does not directly measure individual vertebral joint movement. However, such motions may be estimated by imposing kinematic constraints on a spine model. Kinematic constraints apply interconnections between articulating segments which consequently reduce the degrees of freedom (DOF) and restrict the relative motions. Musculoskeletal models of the spine often rely on kinematic constraints to distribute overall motion to specific levels because skin-surface markers are unable to directly measure the motion of individual intervertebral joints (Lu and O'Connor, 1999; Roux et al., 2002; Andersen et al., 2010; El Habachi et al., 2015; Mason et al., 2016; Rajagopal et al., 2016; Cazzola et al., 2017; Kuo et al., 2018). Applying appropriate kinematic constraints on spine motion can minimize the effect of soft tissue artifacts on segmental kinematics (Lu and O'Connor, 1999), restrict the motion between adjacent segments, and prevent unrealistic intervertebral motions (Leardini et al., 2005; Lu and Chang, 2012). Therefore, appropriate kinematic constraints can generate trajectories for individual vertebral bodies that closely mimic real spinal movement. To our knowledge, most studies using kinematic constraints in musculoskeletal models of the spine have limited the spine to single DOF in each direction (e.g., Lu and O'Connor, 1999; Roux et al., 2002; Anderst et al., 2013; Mason et al., 2016; Bayoglu et al., 2019; Beaucage-Gauvreau et al., 2019), and there has been no examination of how different kinematic constraints might affect the evaluations of spine motion. Fewer constraints (i.e., more DOF) would provide models with more freedom to follow subject-specific spinal motion with less error in tracking the experimental marker data. However, with more DOF, spine kinematics could be more susceptible to movement artifacts causing unrealistic spine angles or discontinuities that prevent a “smooth” motion.

Therefore, the objective of this study was to evaluate the effect of seven different kinematic constraint conditions (allowing three through nine spine DOF) on marker error, smoothness of angular motions and estimated segmental motions when performing inverse kinematics with a thoracolumbar spine model. This information could help identify appropriate kinematic constraints in producing realistic spine motions based on motion analysis data.

## Materials and Methods

### Participants

A convenience sample of seven healthy adult volunteers (4 F, 3 M) was used for this study, drawn from participants in our prior study of the reliability of measuring spine range of motion with an optoelectronic motion analysis system (Mousavi et al., 2018). The mean (±SD) age, height, weight, and BMI of the participants were 42 (±14) years, 172 (±7) cm, 69.6 (±11.1) kg, and 23.3 (±2.3) kg/m^2^, respectively. Exclusion criteria for enrollment included any history of recent back pain, spinal surgery, traumatic vertebral fractures, thoracic deformity, and/or conditions affecting balance, movement, or ability to stand. The study was approved by the Institutional Review Board of Beth Israel Deaconess Medical Center. Written informed consent was obtained from all participants.

### Procedures

At the beginning of each experimental session, age, height, and weight were recorded. For proper placement of retroreflective markers, anatomical landmarks were carefully palpated and marked. Then, retroreflective markers were attached to the skin using double-adhesive tape. Specifically, seven rigid clusters, consisting of four 9.5 mm retroreflective markers, were attached to the skin overlying the T1, T4, T5, T8, T9, T12, and L1 spinous processes. Additional 14-mm markers were placed over the iliac crests, anterior (ASIS) and posterior (PSIS) superior iliac spines, head (using a headband with attached four retroreflective markers), C7, shoulders (bilaterally on the acromion), sternum, clavicles, elbows (lateral epicondyle of the humerus), wrist (radial styloid process), greater trochanter of the femur, lateral and medial aspects of the knee joint, lateral and medial aspects of the ankle joint, posterior heel and first metatarsophalangeal joint (Mousavi et al., 2018). Marker positions during activities were collected using a 10-camera motion analysis system (Vicon Motion Systems, Oxford, UK). All participants were instructed to stand upright and facing forward (as standard anatomical posture) with arms slightly abducted, palms facing anteriorly, and feet shoulder-width apart. Initially, 3-D marker data were collected in the neutral, upright standing posture and static poses held in self-selected maximum flexion, extension, lateral bending, and axial rotation positions for ~5 s each. Participants were then asked to perform three separate dynamic tasks in the following order: spinal flexion–extension, lateral bending (i.e., first left and then right lateral bending), and axial rotation (i.e., first left and then right axial rotation). In each dynamic task, the participants moved to their self-selected maximum ROM in 2 s (following a metronome), returned to neutral in 2 s, moved to their self-selected maximum ROM in the opposite direction in 2 s, and returned to neutral in 2 s. Participants performed this full sequence at least three times for each dynamic task. For the dynamic tasks, participants began in neutral, upright standing with their arms down at their sides, and during the motions, they kept their feet on the ground but were allowed to move their arms as needed to fully complete each motion.

### Development of Kinematic Constraints

#### Ratios of Intervertebral Motion to Overall Spine Motion

For each direction of static and dynamic motion [i.e., flexion–extension (FE), lateral bending (LB), and axial rotation (AR)], we estimated the proportion of each intervertebral level motion from previous work that provided values for FE, LB, and AR in the thoracic and lumbar spines (Table 1). These studies evaluated segmental ranges of motion *in vivo* using a wide variety of techniques, including standard radiographs (Cheng et al., 2016), videofluoroscopy (Wong et al., 2006; Cheng et al., 2016), biplane radiography (Pearcy and Tibrewal, 1984; Shin et al., 2013), MRI (Fujii et al., 2007), CT scans (Fujimori et al., 2012, 2014; Morita et al., 2014), a spinal mouse curvature measurement device (Mannion et al., 2004), motion analysis marker clusters attached to vertebral spinous processes via Kirshner wires (Rozumalski et al., 2008), and an electromagnetic motion analysis system (Willems et al., 1996). We also included reported physiological ROM from the works of White and Panjabi (1978) and Panjabi et al. (1994). The angular motions at each intervertebral joint were converted to ratios relative to overall thoracic or lumbar motion. Because of inconsistencies in the methods used to estimate the intervertebral motions, we used the median value of the reported rotational motion ratio assessed for each level in the current study. The median values determined for thoracic and lumbar spines were then combined and expressed as ratios proportional to overall spine motion (Table 2). Percentage values of intervertebral joint motions to overall thoracic and lumbar spine motions in different studies (i.e., listed studies in Table 1) were shown in Tables 1–4 in Appendix A (Supplementary Material).

**Table 1:** List of all studies used to estimate the intervertebral motion ratios in the thoracic and lumbar spines.

**Study**	**Measurement technique**	**Thoracic**			**Lumbar**		
		**FE**	**LB**	**AR**	**FE**	**LB**	**AR**
Cheng et al. (2016)	Standard bending radiographs/video fluoroscopy				X	X	
Fujii et al. (2007)	MRI						X
Fujimori et al. (2012)	CT-scan			X			
Fujimori et al. (2014)	CT-scan		X				
Mannion et al. (2004)	Spinal Mouse curvature measurement device	X			X		
Morita et al. (2014)	CT-scan	X					
Panjabi et al. (1994)	3D load-displacement curves				X	X	X
Pearcy and Tibrewal (1984)	Biplane radiography					X	X
Rozumalski et al. (2008)	Motion analysis marker clusters attached to vertebral spinous processes via Kirshner wires				X	X	X
Shin et al. (2013)	Biplane radiography						X
White and Panjabi (1978)	Literature review	X	X	X	X	X	X
Willems et al. (1996)	Electromagnetic motion analysis system	X					
Wong et al. (2006)	Video fluoroscopy				X		

*FE, flexion–extension; LB, lateral bending; AR, axial rotation; MRI, magnetic resonance imaging*.

**Table 2:** Ratios of individual-level intervertebral motions to overall spine motion were used to create kinematic constraints in flexion–extension, lateral bending, and axial rotation tasks.

**Joint level**	**Flexion–extension**	**Lateral bending**	**Axial rotation**
L5/S1	0.132	0.037	0.039
L4/L5	0.155	0.082	0.040
L3/L4	0.150	0.104	0.040
L2/L3	0.160	0.101	0.038
L1/L2	0.118	0.081	0.036
T12/L1	0.037	0.067	0.020
T11/T12	0.039	0.073	0.035
T10/T11	0.039	0.057	0.060
T9/T10	0.030	0.050	0.073
T8/T9	0.019	0.045	0.085
T7/T8	0.017	0.051	0.087
T6/T7	0.015	0.041	0.079
T5/T6	0.015	0.036	0.078
T4/T5	0.009	0.038	0.074
T3/T4	0.016	0.046	0.070
T2/T3	0.024	0.045	0.074
T1/T2	0.028	0.046	0.072

*The value in each cell is the median value of the rotational motion ratio assessed for each intervertebral level (based on all studies listed in Table 1)*.

#### Kinematic Constraints Employed in Models

The ratios presented in Table 2 were applied as kinematic constraints in subject-specific models. The ratios shown in Table 2 reduced the overall spine kinematics to a single DOF in each orthogonal direction of spine motion or three DOF overall. Segmental motion data from our prior study suggests that some segments of the spine act differently than others during certain motions (Mousavi et al., 2018). For example, the upper thorax may extend during full lumbar flexion in some individuals, while the lumbar and thoracic spines may display different behaviors, particularly during axial rotation motions. Thus, we created multiple kinematic constraint conditions allowing three through nine DOF, as shown in Table 3. The total spine DOF in models with kinematic constraints refers to the number of independent rotational coordinates, which link the motions within specific sections of the spine. In these models, motion occurs at all levels, but the individual rotations at each level are dependent on the corresponding independent coordinate. For example, for the 3DOF model, the spine has only three independent coordinates, which describe FE, LB, and AR for the entire spine (T1-S1), and the motion of each intervertebral joint is defined as a proportion of the overall motion of the spine. However, in the 4DOF model, the FE motion of the spine has two independent coordinates, applied to sections T1-T9 and T9-S1, respectively, but still just a single independent coordinate in each of the LB and AR directions of motion (see Table 3). We also included a baseline condition (i.e., with no kinematic constraint), allowing 51 combined rotational DOF for the 17 thoracolumbar joints. The ratios of individual-level intervertebral motions to the overall spine for all kinematic constraints (3-9DOF) are shown in Table 1 of Appendix B (Supplementary Material).

**Table 3:** Summary of kinematic constraint conditions tested, indicating the total spine DOF and DOF in each rotational direction, plus the spine sections for each DOF and rotational direction.

**Condition/Total DOF**	**FE DOF/Spine sections**	**LB DOF/Spine sections**	**AR DOF/Spine sections**
3DOF	1 [T1-S1]	1 [T1-S1]	1 [T1-S1]
4DOF	2 [T1–T9, T9-S1]	1 [T1-S1]	1 [T1-S1]
5DOF	2 [T1–T9, T9-S1]	2 [T1-L1, L1-S1]	1 [T1-S1]
6DOF	2 [T1–T9, T9-S1]	2 [T1-L1, L1-S1]	2 [T1-L1, L1-S1]
7DOF	2 [T1–T9, T9-S1]	2 [T1-L1, L1-S1]	3 [T1–T9, T9-L1, L1-S1]
8DOF	2 [T1–T9, T9-S1]	3 [T1–T5, T5-L1, L1-S1]	3 [T1–T9, T9-L1, L1-S1]
9DOF	3 [T1–T9, T9-L1, L1-S1]	3 [T1–T5, T5-L1, L1-S1]	3 [T1–T9, T9-L1, L1-S1]

*FE, flexion–extension; LB, lateral bending; AR, axial rotation; DOF, degrees of freedom*.

### Subject-Specific Modeling and Inverse Kinematics Using OpenSim

For each participant, we created a subject-specific musculoskeletal model based on our previously validated models of the thoracolumbar spine (Bruno et al., 2015, 2017). Models were scaled according to subject height and weight, with body segment lengths and spine curvature adjusted based on marker data recorded in a neutral static standing position (Burkhart et al., 2020). For each subject, we created one model without kinematic constraints applied (allowing 51 spine DOF). Seven additional models were created with the seven sets of kinematic constraints described earlier to limit spine motion to 3–9DOF. For each model and activity measured, we performed inverse kinematics (IK) in OpenSim [version 3.3; Delp et al. (2007)] to compute the joint angles that would best match the model to the measured marker positions. In all models, FE, LB, and AR are independent motions and are determined simultaneously in the inverse kinematics analysis. The marker positions used for IK analyses were first low-pass filtered (6 Hz, fourth-order Butterworth filter, bidirectional). After completing IK analyses, OpenSim generated motion files containing the relative angles between adjacent vertebrae in each direction of motion (FE, LB, AR). The kinematic results were additionally used to evaluate Euler angles for particular spine segments of interest.

### Outcome Measures and Statistical Analyses

The primary outcomes for this study were: (1) root mean square (RMS) error of recorded vs. tracked spine markers; (2) lag-one autocorrelation coefficient of the segmental (i.e., T1-T5, T5-T9, T9-L1, L1-S1) angular motions for each primary direction of tasks (FE, LB, and AR); (3) maximum ROM of intervertebral joint angles across three dynamic tasks (flexion–extension, lateral bending, axial rotation) for all combinations of kinematic constraints and directions of motion (i.e., FE, LB, AR); and (4) segmental spine angles in the static range of motion trials for four spine sections (i.e., T1–T5, T5–T9, T9-L1, L1-S1). Statistical analyses for the first three primary outcomes were performed using JMP Pro 15 (SAS, Cary, NC), using the restricted maximum likelihood method, with a statistical significance level of 0.05. Analyses of static segmental spine angles were performed in Stata/IC 13.1 (StataCorp LP, College Station, TX).

#### RMS Error of Spine Markers

We defined the RMS error of spine markers for each subject within each task and kinematic constraint as the square root of the sum of marker errors squared (measured vs. model) divided by the number of markers (e.g., 28 markers = 7 clusters × 4 markers on each cluster). For each task, we calculated the mean ± SD of RMS error of markers for each set of kinematic constraints [baseline or no constraint (51DOF) and seven sets of kinematic constraints (3–9DOF)]. We used a two-way repeated-measures analyses of variance (ANOVA) to examine the effects of *Task* and *Constraint* on RMS error of spine markers. Because the interaction effect *Task* × *Constraint* was not statistically significant, we simplified the model by employing separate one-way ANOVA for each task to assess the effect of *Constraint* (as an independent variable) on RMS marker error. Significant effects were followed by pairwise comparisons [Tukey's honest significance difference (HSD)] and simple effects testing, where relevant.

#### Lag-One Autocorrelation Coefficient of the Segmental Angular Motions

The lag-one autocorrelation coefficient reflects the correlation between values that are one time-step apart and thereby quantifies how much a point in a signal is predictable based on the previous point. Therefore, it can be used as an index to assess the smoothness of the angular motions. We computed the lag-one autocorrelation coefficients of the segmental angular motions for each primary direction of tasks (i.e., FE for flexion–extension, LB for lateral bending, and AR for axial rotation). We performed logit transformation on the autocorrelation coefficients as they were between 0 (least smooth) and 1 (smoothest) and had exhibited a skewed distribution. A one-way repeated measure of ANOVA for each primary task direction was applied to determine the effect of different kinematic constraints (as an independent variable) on the transformed autocorrelation coefficients. Statistically significant effects were followed by pairwise comparisons (Tukey's HSD) and simple effects testing, where relevant.

#### Maximum ROM of Intervertebral Joint Angles

To determine if the intervertebral joint angles are in a physiologically realistic range, we calculated the maximum ROM of the 17 intervertebral joint angles (i.e., T1/T2, T2/T3, …, L5/S1) grouped in three spine regions (T1–T9, T9-L2, L2-S1), for all combinations of kinematic constraints [3–9DOF and no constraint (51DOF)] and directions of motion (FE, LB, AR). Note that the maximum ROM for each intervertebral joint in each direction of motion was defined using the maximum angles recorded across all three dynamic tasks (i.e., maximum flexion angle–maximum extension angle, maximum left lateral bending angle – maximum right lateral bending angle, maximum left axial rotation angle – maximum right axial rotation angle). These ROMs were compared with the estimated limits of normal ROMs for intervertebral joints presented by White and Panjabi (1978). Because joints within the same part of the spine are expected to have similar ROMs, we combined results within three regions of the spine to simplify comparisons. Specifically, boxplots of ROMs created for thoracic (T1-T2 through T8-T9), thoracolumbar (T9-T10 through L1-L2), and lumbar (L2-L3 through L5-S1) joints and compared to the corresponding expected ranges of joint ROM.

#### Segmental Spine Angles in the Static Range of Motion Trials

To evaluate whether various kinematic constraints applied to a spine model allow the model to reasonably match measured positions, we compared segmental angles calculated directly from marker cluster orientations to those produced in the model after IK analysis. Marker data were collected for six static standing trials, specifically, while subjects held the position at their full ranges of motion for flexion, extension, left and right lateral bending, and left and right axial rotation. As previously described (Mousavi et al., 2018), Euler angles were calculated between marker clusters to provide rotations for segments T1–T5, T5–T9, T9-L1, and L1-S1.

Agreement between model segmental angles and corresponding cluster angles was evaluated with intra-class correlation coefficients (ICCs) and RMS differences. ICC results were deemed weak (ICC < 0.5), moderate (0.5 ≤ ICC < 0.75), good (0.75 ≤ ICC < 0.9), or excellent (0.9 ≤ ICC) (Koo and Li, 2016), including their 95% CIs. One-way repeated measures ANOVAs were used to examine whether model angles were different from the corresponding cluster angles. Analyses were performed separately for flexion–extension, lateral bending, and axial rotation angles, grouping all segments and trials to provide an overall evaluation of agreement of segmental angles for each direction of spine motion.

## Results

### RMS Error of Spine Markers

**Figure 1:**
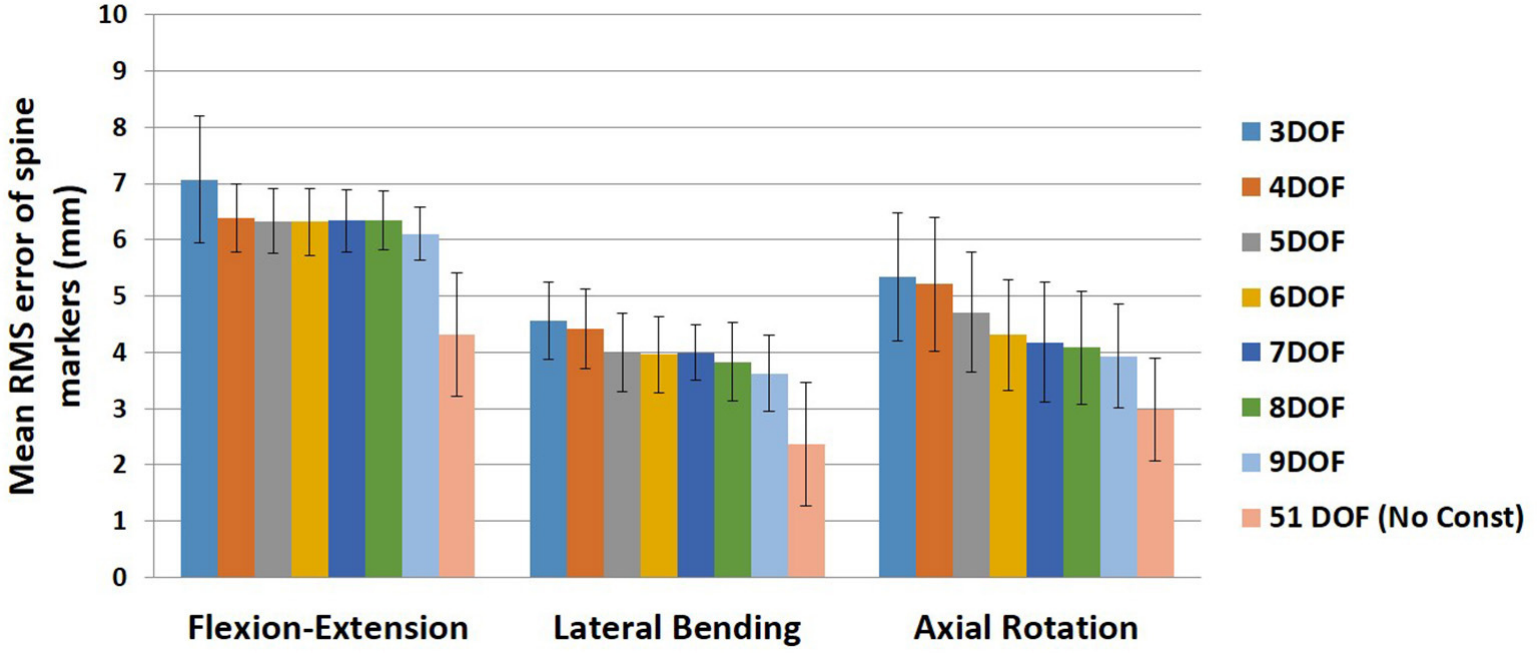
Mean RMS error of spine markers for all kinematic constraints (3–9DOF) and no constraint condition (51DOF) in all tasks (flexion–extension, lateral bending, and axial rotation). Error bars indicate the SDs. RMS, root mean square; DOF, degrees of freedom.

The mean and standard deviation of RMS error of spine markers for different sets of kinematic constraints [3–9DOF and no constraint (51DOF)] in three tasks are summarized in Figure 1. Our first statistical analysis demonstrated that although there were significant main effects of *Task* (*p* < 0.0001) and *Constraint* (*p* < 0.0001) on RMS error of spine markers, we did not observe a *Task* × *Constraint* interaction effect (*p* ~ 0.499). The Tukey HSD *post-hoc* analyses showed that RMS error of spine markers was significantly different for all pairs of different tasks [i.e., flexion–extension greater than lateral bending (*p* < 0.0001), flexion–extension greater than axial rotation (*p* < 0.0001), and lateral bending smaller than axial rotation (*p* < 0.0001)]. Additionally, the results of our second statistical analysis revealed that a significant effect of *Constraint* within each task was observed for RMS marker errors. All other pairwise comparisons for the constraints within each task are presented in Table 4. Across all tasks, the RMS errors of spine markers for 51DOF were significantly lower than other constraints (*p*-values ≤ 0.0002).

**Table 4:** Significant *post-hoc* pairwise comparisons (Tukey's HSD) of kinematic constraints for RMS error of spine markers in each task shown in Figure 1.

**Kinematic constraint**	**Flexion-extension**	**Lateral bending**	**Axial rotation**
3DOF	4–9DOFs and 51DOF	5–9DOFs and 51DOF	6–9DOFs and 51DOF
4DOF	3DOF and 51DOF	8–9DOF and 51DOF	6–9DOFs and 51DOF
5DOF	3DOF and 51DOF	3DOF and 51DOF	9DOF and 51DOF
6DOF	3DOF and 51DOF	3DOF and 51DOF	3–4DOFs and 51DOF
7DOF	3DOF and 51DOF	3DOF and 51DOF	3–4DOFs and 51DOF
8DOF	3DOF and 51DOF	3–4DOF and 51DOF	3–4DOFs and 51DOF
9DOF	3DOF and 51DOF	3–4DOFs and 51DOF	3–5DOFs and 51DOF

*The constraint(s) in each cell are the constraint(s) significantly different from the corresponding constraint listed in the first column. HSD, honest significance difference; RMS, root mean square; DOF, degrees of freedom*.

For the flexion–extension, there were no differences in RMS errors between any pair of kinematic constraints with 4–9DOF, whereas the RMS errors for kinematic constraints with 4–9DOF were all significantly lower than the 3DOF model (*p*-values ≤ 0.0291) and higher than the 51DOF model (*p*-values < 0.0001). In the lateral bending, there were significant differences between models with lower and higher DOF [i.e., 3DOF >5–9DOF, and 4DOF >8–9DOF, (*p*-values < 0.05); Table 4], but there were no significant differences between any sequential pairs of constraints from 5 to 9DOF. Further, for axial rotation, 6DOF or higher kinematic constraints improved the RMS error of spine markers vs. 3–4DOF. However, no differences between any constraints from 6 to 9DOF were found.

### Lag-One Autocorrelation Coefficient of the Angular Motions

**Figure 2:**
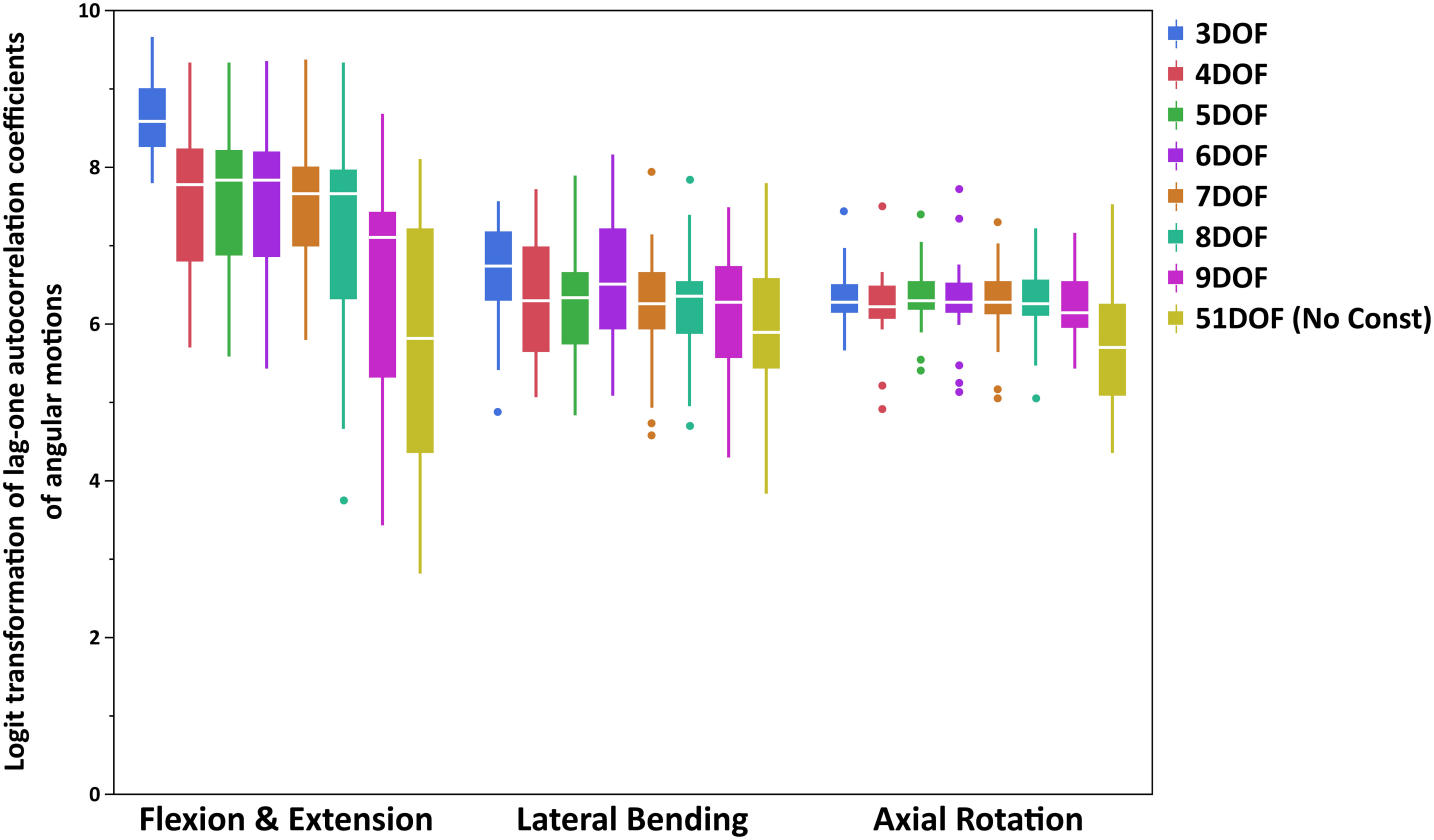
Boxplot of logit transformed lag-one autocorrelation coefficients of angular motions for each primary direction of tasks (FE, LB, and AR). Note that logit transformed autocorrelation values extend from 0 *(least smoothness*) to 10 (*smoothest*). FE, flexion–extension; LB, lateral bending; AR, axial rotation.

The lag-one autocorrelation coefficients varied significantly by *Task* (*p* < 0.0001) and *Constraint* (*p* < 0.0001). The box plot of logit transformed autocorrelation coefficients for different tasks and constraints is shown in Figure 2, with larger values indicative of “smoother” joint motion [transformed autocorrelation values on y-axis extend from 0 (*least smoothness*) to 10 (*smoothest*)]. For the flexion–extension task, the lag-one autocorrelation coefficients for all but the 9DOF constraint showed significantly smoother motion than the 51DOF model (*p* < 0.0001; Table 5). Additionally, the motion was significantly smoother for the 3DOF model than for all other constraints. The lag-one autocorrelation coefficient did not differ between sequential constraints for flexion–extension, except for smoother motion in the 3DOF model than the 4DOF model (*p* = 0.0138). For lateral bending tasks, the lag-one autocorrelation coefficient differed for three pairs of constraints [3DOF vs. 9DOF, 3DOF vs. 51DOF, and 6DOF vs. 51DOF (*p* < 0.05)], but the differences in lag-one autocorrelation coefficients between sequential constraints were not significant. Finally, motion during all axial rotations was smoother for all constrained models than the 51DOF model (*p* < 0.0001) but was similar for all other constraint comparisons.

**Table 5:** Pairwise comparisons (Tukey's HSD) of kinematic constraints for lag-one autocorrelation coefficients of angular motions for each primary direction of tasks (FE, LB, and AR).

**Kinematic constraint**	**Flexion-extension**	**Lateral bending**	**Axial rotation**
3DOF	4–9DOFs and 51DOF	9DOF and 51DOF	51DOF
4DOF	3DOF, 9DOF and 51DOF	NS	51DOF
5DOF	3DOF, 9DOF and 51DOF	NS	51DOF
6DOF	3DOF, 9DOF and 51DOF	51DOF	51DOF
7DOF	3DOF and 51DOF	NS	51DOF
8DOF	3DOF and 51DOF	NS	51DOF
9DOF	3–6DOFs	3DOF	51DOF

*The constraint(s) in each cell is the constraint(s) that were significantly different from the corresponding constraint in the first column. NS, no significant differences; HSD, honest significance difference; FE, flexion–extension; LB, lateral bending; AR, axial rotation; DOF, degrees of freedom*.

### Maximum ROM of Intervertebral Joint Angles

**Figure 3:**
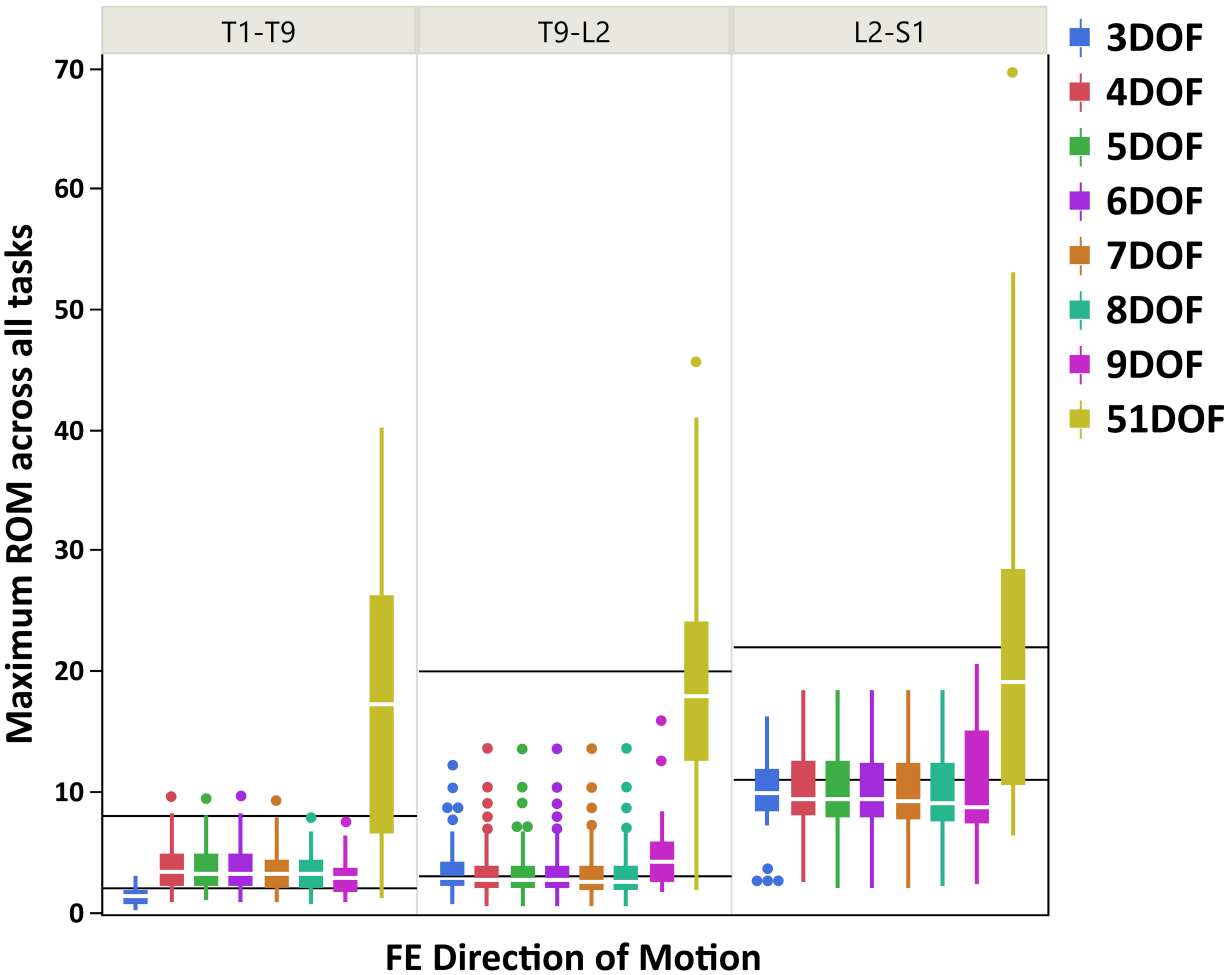
Boxplot of maximum ROM of 17 intervertebral joint angles, grouped in three spine regions [i.e., “T1-T9”: T1/T2 to T8/T9; “T9-L2”: T9/T10 to L1/L2; “L2-S1”: L2/L3 to L5/S1)], for all kinematic constraints [3–9DOF and no constraint (51DOF)] in FE direction of motion. The black lines horizontally overlaid on each panel plot are minimum and maximum ROM of intervertebral joints for the corresponding spine regions in FE direction of motion, reported by White and Panjabi (1978). ROM, range of motion; DOF, degrees of freedom; FE, flexion–extension.

**Figure 4:**
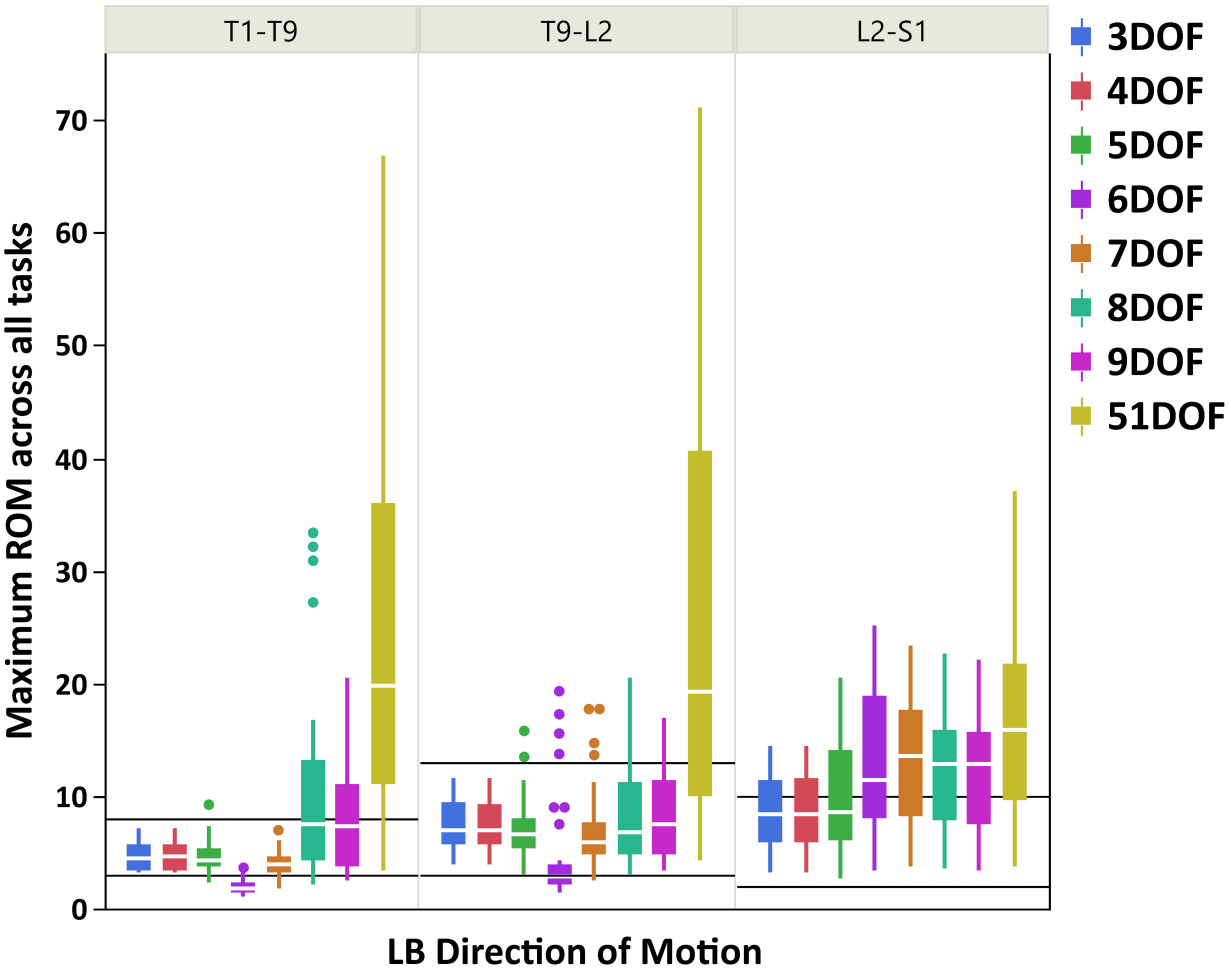
Boxplot of maximum ROM of 17 intervertebral joint angles, grouped in three spine regions [i.e., “T1-T9”: T1/T2 to T8/T9; “T9-L2”: T9/T10 to L1/L2; “L2-S1”: L2/L3 to L5/S1], for all kinematic constraints [3–9DOF and no constraint (51DOF)] in LB direction of motion. The black lines horizontally overlaid on each panel plot are minimum and maximum ROM of intervertebral joints for the corresponding spine regions in LB direction of motion, reported by White and Panjabi (1978). ROM, range of motion; DOF, degrees of freedom; LB, lateral bending.

**Figure 5:**
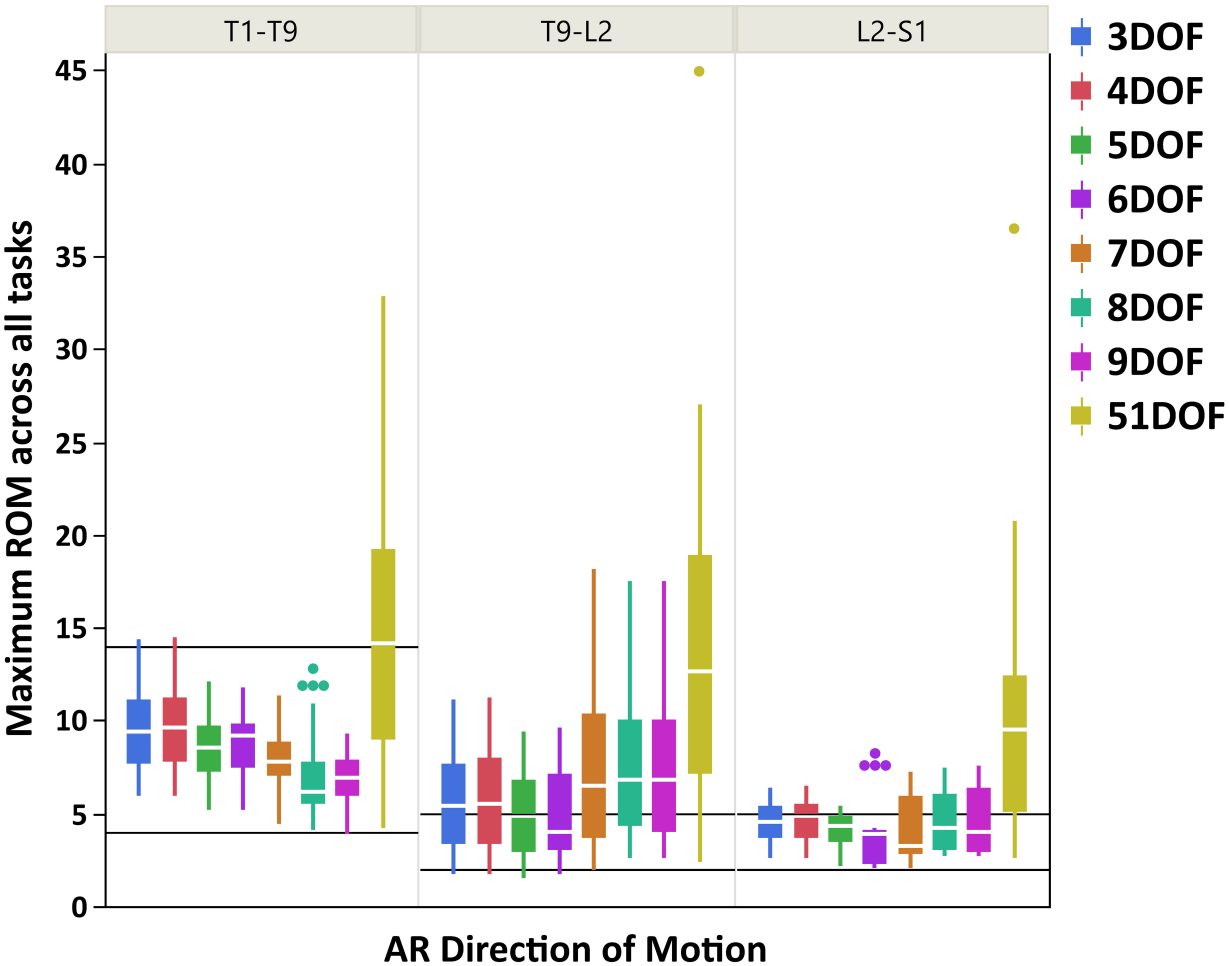
Boxplot of maximum ROM of 17 intervertebral joint angles, grouped in three spine regions [i.e., “T1-T9”: T1/T2 to T8/T9; “T9-L2”: T9/T10 to L1/L2; “L2-S1”: L2/L3 to L5/S1], for all kinematic constraints [3–9DOF and no constraint (51DOF)] in AR direction of motion. The black lines horizontally overlaid on each panel plot are minimum and maximum ROM of intervertebral joints for the corresponding spine regions in AR direction of motion, reported by White and Panjabi (1978). ROM, range of motion; DOF, degrees of freedom; AR, axial rotation.

Boxplots of maximum ROM of intervertebral joint angles (grouped in three spine regions: thoracic: T1–T9, thoracolumbar: T9-L2, lumbar: L2-S1) for all kinematic constraints [3–9DOF and no constraint (51DOF)] and directions of motion (FE, LB, AR) are depicted in Figures 3 (FE), 4 (LB), and 5 (AR). To further compare our findings with the results reported in White and Panjabi (1978), we overlaid solid black horizontal lines in Figures 3–5, representing the minimum and maximum ROM of intervertebral joints in each spine region and direction of motion. The corresponding values of black horizontal lines in each panel plot in Figures 3–5 have been calculated based on the results in Table 2 of conducted study by White and Panjabi (1978). Readers of this paper are referred to Tables 1, 2 in Appendix C (Supplementary Material) for the range of maximum ROM of all individual intervertebral joint angles across four tasks (i.e., flexion, extension, lateral bending, axial rotation) for each combination of kinematic constraints [3–9DOF and no constraint (51DOF)] and direction of motion (FE, LB, AR). In Tables 1, 2 in Appendix C (Supplementary Material), the flexion–extension tasks were divided into flexion and extension separately.

As seen in Figures 3–5, on average, the range and interquartile ranges of the maximum ROM of intervertebral joint angles for 51DOF are often much larger than with constraints and findings in White and Panjabi (1978). For FE direction of motion (Figure 3), the medians of the ROM for constraints with 4–9DOF were close to each other and were largely within the reported range of White and Panjabi (1978) for the thoracic (T1–T9) region, while 3DOF underestimated the expected ROM. The medians for most constraints fell slightly below the reported range in the thoracolumbar region (T9-L2) and more so for the lumbar region (L2-S1), but the maximum ROMs found fell within the reported range. For LB direction (Figure 4), medians with 3–5DOF fell within the range reported by White and Panjabi (1978) in all spinal regions, although the maximum ROMs exceeded the maximum reported ROM in the lumbar region (L2-S1). Median LB ROMs with 6–51DOF exceeded the maximum reported ROM from White and Panjabi for the lumbar region (L2-S1). For AR direction (Figure 5), the medians of the ROM for all constraints were within the ranges reported by White and Panjabi for thoracic (T1–T9) and lumbar (L2-S1) regions. But only 5 and 6DOF had medians within the range for the thoracolumbar (T9-L2) region, although the maximum ROMs exceeded the maximum reported ROM. Overall, it seems that constraints 4DOF through 9DOF all produced similar and largely reasonable ROMs in FE, and similarly constraints 3DOF through 5DOF for LB, and constraints 5DOF and 6DOF for AR.

### Segmental Spine Angles in the Static Range of Motion Trials

The segmental angle RMS differences ranged from 9.1 to 10.0° for flexion–extension, 8.4–10.5° for lateral bending, and 10.3–12.1° for axial rotation angles (Table 6). Models with kinematic constraints had moderate to good ICCs for flexion–extension and lateral bending, though weak to moderate ICCs for axial rotation. The statistical results of ANOVAs indicated a significant effect of the angle evaluation approach (marker cluster orientations, models with kinematic constraints, model without kinematic constraints) for flexion–extension angles (*p* = 0.022), but not for lateral bending or axial rotation angles. However, *post-hoc* testing showed that none of the flexion–extension segmental angles evaluated by the models differed from those evaluated from marker cluster orientations (i.e., any differences were between different models).

**Table 6:** RMSD and ICCs for segmental spine angles from models with various spine DOFs vs. segmental angles calculated directly from the corresponding marker clusters.

**Kinematic constraints** **(DOF)**	**Flexion-extension**		**Lateral bending**		**Axial rotation**	
	**RMSD**	**ICC (95% CI)**	**RMSD**	**ICC (95% CI)**	**RMSD**	**ICC (95% CI)**
3	9.3	0.779 (0.712–0.833)	8.5	0.787 (0.720–0.839)	11.5	0.514 (0.391–0.612)
4	9.1	0.796 (0.732–0.846)	8.5	0.787 (0.720–0.839)	11.6	0.516 (0.392–0.621)
5	9.4	0.795 (0.730–0.845)	9.0	0.776 (0.707–0.830)	11.8	0.470 (0.340–0.582)
6	9.2	0.803 (0.740–0.852)	10.1	0.728 (0.647–0.794)	11.2	0.531 (0.410–0.633)
7	9.6	0.784 (0.716–0.837)	9.4	0.766 (0.693–0.823)	11.4	0.530 (0.409–0.633)
8	10.0	0.783 (0.716–0.836)	10.5	0.704 (0.617–0.774)	12.1	0.515 (0.391–0.620)
9	9.8	0.795 (0.730–0.845)	10.3	0.710 (0.624–0.779)	11.7	0.541 (0.422–0.642)
51	9.8	0.789 (0.723–0.841)	9.1	0.785 (0.718–0.838)	10.3	0.687 (0.596–0.761)

*RMSD, root mean square differences; ICC, intraclass correlation coefficient; DOF, degrees of freedom*.

## Discussion

This study explored the effect of different kinematic constraints on model performance during IK analysis of the thoracolumbar spine. This novel examination addresses a gap in knowledge regarding the influence of kinematic constraints with multiple DOFs on spine motion assessments. Most kinematic constraints previously imposed in musculoskeletal modeling have assumed a single DOF for each direction of spinal bending or rotation. We evaluated the effect of kinematic constraints with four main outcome measurements, including (1) RMS error of spine markers (measured vs. model); (2) lag-one autocorrelation coefficients to quantify the smoothness of angular motions; (3) maximum ROMs of 17 intervertebral joint angles, grouped in three spine regions, and whether they are in a physiologically realistic range; and (4) segmental spine angles in the static range of motions trials to verify if the spine models with different kinematic constraints reasonably match measurements directly from marker clusters.

### RMS Error of Spine Markers

**Figure 6:**
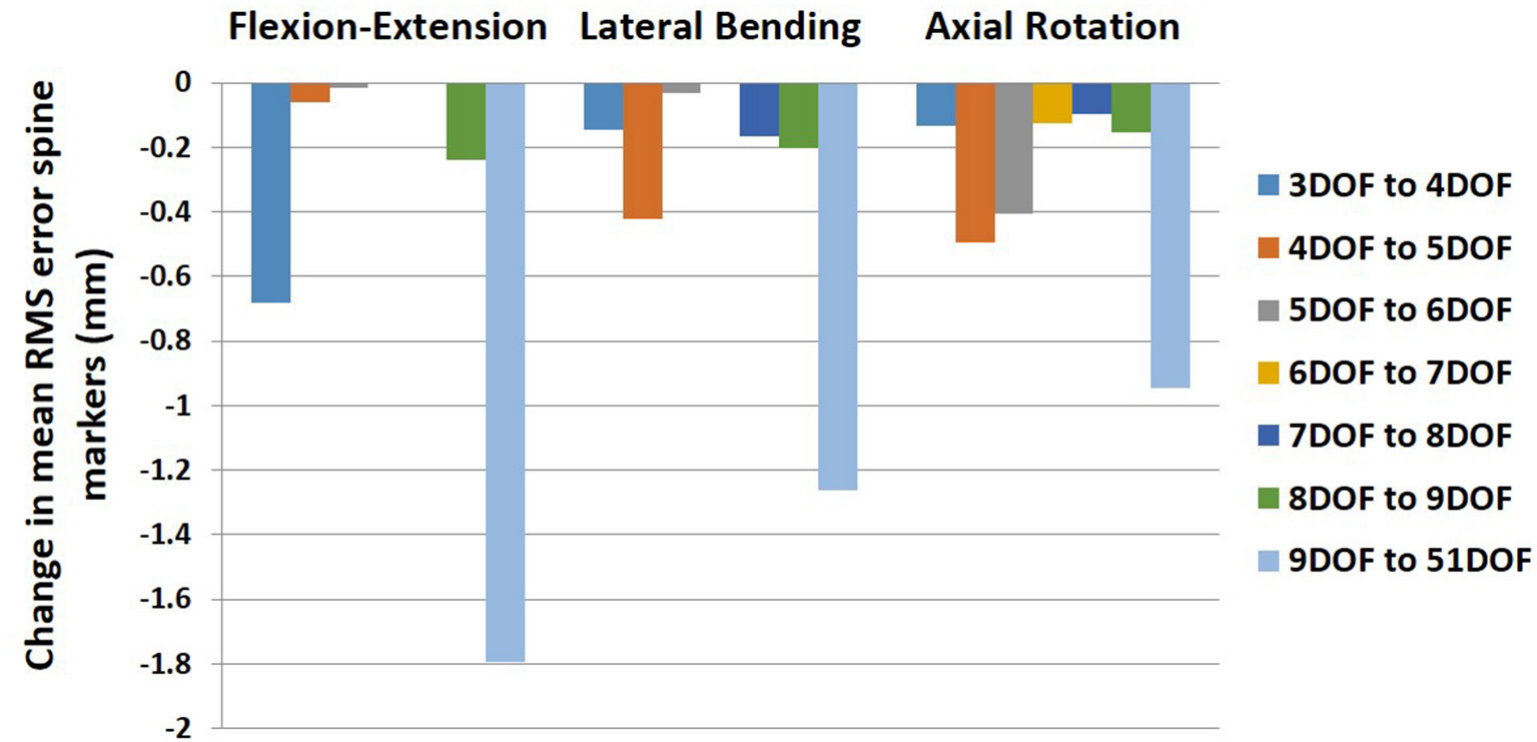
Change in mean RMS error of spine markers for all pairs of sequential kinematic constraints in all tasks (flexion–extension, lateral bending, and axial rotation). RMS, root mean square.

On average, the RMS error of spine markers for flexion–extension was higher than lateral bending and axial rotation across all kinematic constraints and no-constraint conditions. It is possible that the skin-surface spine markers are more sensitive to flexion–extension motions as they are largely aligned with the spine in the sagittal plane. RMS error of spine markers in axial rotation appeared to be slightly larger than lateral bending, perhaps because the more complex nature of spine movement in axial rotation (i.e., combination of rotational and coupled bending movement) causes a larger difference between the modeled and experimental position coordinates. Any differences in RMS error of spine markers should be checked for practical significance, as they might not be meaningful if less than the precision of the optoelectronic motion capture system. Merriaux et al. (2017) investigated the positioning performance of the Vicon motion capture system. They reported that the optimal position performance depends on the sampling rate of the system and the marker size, but with optimized performance, the mean absolute error for static and dynamic tasks could be as low as 0.15 and 0.3 mm, respectively. Thus, to define the practical importance of the RMS error of spine markers in our study, we compared the reduction in RMS error of markers between pairs of sequential constraints with the dynamic error estimate of 0.3 mm. Based on this comparison (Figure 6), no meaningful improvements in RMS error of spine markers were observed for the flexion–extension task after 4DOF. For lateral bending and axial rotation tasks, meaningful improvements in mean RMS error of spine markers were observed when changing from 4 to 5DOF, and for axial rotation, an additional meaningful improvement was seen in changing from 5 to 6DOF. In sum, depending on the type of the task, the results suggest that kinematic constraints with 4, 5, or 6 DOF may be beneficial, but additional DOF above 6 would not produce meaningful improvements on the RMS error of spine markers. Figure 6 also includes the change in mean RMS error of spine markers from 9 to 51DOF, showing meaningful improvements in all tasks. It is expected that 51DOF would produce the smallest RMS error of spine markers among the conditions tested since the spine model with higher DOF can theoretically better adapt to the variation in subject motion. However, it is worth noting that the 1.8 mm improvement in RMS error (seen in flexion–extension) between the 9DOF and the 51DOF model comes by adding 42 more DOF. Thus, each additional DOF improves the RMS error by an average of 0.04 mm, seemingly well below any meaningful value.

### Lag-One Autocorrelation Coefficient of the Angular Motions

The lag-one autocorrelation coefficients showed that kinematics in axial rotation were smoother with any constraint from 3 to 9DOF than without constraints (51DOF). This could also be visually inspected from Figure 3 in Appendix B (Supplementary Material), where the averaged angular motions of T9-L1 and L1-S1 spine segments for 51DOF were much less smooth than with kinematic constraints. Similarly, kinematics in flexion–extension was smoother with any constraint from 3 to 8DOF than 51DOF, where these findings suggested that moving from 2 to 3 FE DOF would lead the spinal angular motions to be less smooth compared to no-constraint conditions. For the lateral bending, in addition to 3DOF, the 6DOF constraint produced smoother angular motions compared with the 51DOF. This might be partially explained through interactions between LB and AR motions, as the 6DOF constraint is the only constraint with two AR DOF. Overall, the 3DOF constraint produced the smoothest motions during flexion–extension and lateral bending, and this is theoretically expected as the 3DOF constraint has the lowest DOF compared to all other constraints. The constraints 4–6DOF showed similar smoothness behavior in flexion–extension, and all produced smoother kinematics than the 9DOF constraint. Notably, flexion–extension tasks showed higher RMS error of spine markers and higher autocorrelation values (i.e., higher level of smoothness) than lateral bending or axial rotation tasks, indicating limiting DOF in flexion–extension may improve kinematic smoothness at the expense of accuracy. Overall, the lag-one autocorrelation results indicate that constraints between 4 and 6DOF produced similar levels of smoothness for angular motions across all tasks and generally improved smoothness compared to 9DOF or unconstrained models.

### Maximum ROM of Intervertebral Joint Angles

The main objective of this section is to assess whether various constraint conditions produce maximum joint ROMs that fall within a physiologically reasonable range. Results in Figures 3–5 demonstrated that maximum ROMs without constraints (51DOF) were notably larger than with constraints for all directions of motion and all regions of the spine and exceeded reasonable physiological ranges. Thus, performing inverse kinematics without kinematic constraints (51DOF) is unsuitable for characterizing *in vivo* spine motion.

On average, results in Figure 3 suggested that for the FE direction of motion, kinematic constraints 4–9DOF produced quite similar maximum ROMs across all tasks and in all spine regions. The maximum ROM with these constraints did not exceed a physiologically reasonable value. However, the ROMs tended to underestimate the expected values of White and Panjabi (1978) in the thoracolumbar and especially the lumbar regions of the spine. It is possible that the participants tested for this study did not reach their maximum lumbar ROM during the flexion–extension task, which could explain this result. Nonetheless, our results revealed higher maximum ROM of the lumbar intervertebral joints than the thoracic and thoracolumbar joints, in agreement with the trend of expected ROM across different parts of the spine (White and Panjabi, 1978).

For LB direction of motion, larger maximum ROMs were observed for the thoracolumbar and lumbar regions than the thoracic region, consistent with the expected ROMs reported by White and Panjabi (1978). Figure 4 also indicated that ROMs for kinematic constraints with 3–5DOF were largely within the physiologically acceptable range as reported by White and Panjabi (1978), except that the maximum ROMs with these kinematic constraints could exceed the expected maximum ROM of the lumbar region. The 6DOF constraint uniquely appeared to underestimate LB ROM in the thoracic spine. Interestingly, 8 and 9DOF kinematic constraints demonstrated much higher variability in the thoracic region than 3–7DOF constraints. This is likely because these kinematic constraints have three LB DOF, while the other constraints have only one or two LB DOF.

For AR direction of motion, the maximum ROMs for kinematic constraints with 3–6DOF were largely within the reported physiologically acceptable range from White and Panjabi (1978) for thoracic and lumbar regions. However, all constraints tended to overestimate the expected ROM in the thoracolumbar region, where the lowest differences belonged to constraints with 5 and 6DOF. Our results generally found thoracolumbar joint ROMs to fall between thoracic and lumbar values, while the expected thoracolumbar ROM from White and Panjabi (1978) was identical to the lumbar values. Nonetheless, the maximum ROM of the thoracic intervertebral joints was notably larger than the lumbar intervertebral joints, following the expected trend reported in White and Panjabi (1978). Overall, it seems that kinematic constraints 5 and 6DOF produced the most consistent AR joint ROM results compared with the expected values of White and Panjabi (1978).

In sum, our findings suggest that the maximum ROM predicted using 4DOF through 6DOF constraints were within the physiologically acceptable range for the majority of spine regions, and directions of motions, based on comparison with the representative values reported in White and Panjabi (1978). The 5DOF kinematic constraint on balance seemed to provide the best and most stable result for all directions of motion. The 4DOF median ROM for thoracolumbar AR slightly exceeded the expected maximum value but was, in fact on quite similar to the 5DOF value. The 6DOF produced unusual LB motion patterns, underestimating expected thoracic ROM while exceeding expected lumbar ROM. It is important to note that the expected ranges of ROM reported by White and Panjabi (1978) are representative values based on a review of experimental data and expert opinion, thus should not be considered a “gold standard.” The observed discrepancies between our results and the expected ROM of White and Panjabi (1978) are not out of line with discrepancies between that and various other studies (e.g., Pearcy and Tibrewal, 1984; Pearcy et al., 1984; Li et al., 2009) reporting intervertebral joint ROM. These discrepancies can be explained by implementing different methodological approaches to measure the intervertebral joint angles and evaluate the ranges of motion.

### Segmental Spine Angles in the Static Range of Motion Trials

Overall, the results suggest that all models predicted similar segmental angles to those calculated directly from the marker data. Introducing different kinematic constraints in the models had no discernable effect on the RMS differences or ICCs for flexion–extension and lateral bending angles. For axial rotations, the models with kinematic constraints had uniformly higher RMS differences than the unconstrained model and lower ICCs (though generally with some overlap in the confidence intervals). In sum, all models with kinematic constraints appear equally valid, and adding more DOF did not improve performance, matching segmental angles calculated directly from spine marker clusters. It is important to note that the segmental angles calculated directly from markers do not represent a gold standard measurement of the underlying spine motion. Thus, this analysis does not directly address the accuracy of the models in predicting underlying spine motion but shows that model-predicted values should have similar validity as marker-based spine motion directly.

### Study Limitations

A few potential limitations should be noted for this study. First, the study had a small sample size of seven healthy participants, so it is possible that the range of healthy normal spine motions was not fully represented. Moreover, it is unclear to what extent our results would be applicable for patients with spinal disorders. Therefore, further studies with larger and more heterogeneous sample sizes are needed to verify the generalizability of our findings. Second, during the data collection, the retroreflective markers were placed on bony landmarks by multiple experimenters, consequently adding some errors to our study (Della Croce et al., 1999). Third, we used marker sets with clusters in our study, and the generalizability to the use of different marker sets was not examined. Fourth, passive structure contributions such as spinal ligaments and intervertebral discs are not currently considered, nor are muscle forces or vertebral loading. Additionally, vertebral joints were modeled as ball joints with 3DOF and did not explicitly introduce within joint motion coupling or allow for any joint translational motion. Coupled intervertebral motion has been established in cadaveric testing studies (Panjabi et al., 1976; Gardner-Morse and Stokes, 2004), including between axial and lateral bend rotational motions, but it remains unclear how to characterize such coupling in kinematic constraints for kinematics analyses appropriately. However, as the FE, LB, and AR directions of motion remained independent in all analyses, the models can adopt coupled motions to best match the marker data. Thus, the model neither requires nor precludes coupled motion. A few studies have proposed optimization methods to adjust individual-level spinal kinematics to minimize passive structure forces–called force-dependent kinematics (Meng et al., 2015; Ignasiak et al., 2016) or minimize muscle mass and spine loading (Shojaei et al., 2015). These approaches may ultimately help to assign vertebral kinematics more accurately than kinematic constraints uniquely. They may even incorporate coupling and translational motion, but they pose significant challenges, including the need to characterize passive structure properties accurately and increased computational cost. Fifth, there are numerous possibilities for reasonable kinematic constraint conditions beyond the seven examined here, both in terms of the ratios used and the number and distribution of spinal DOF. There is no assurance that the kinematic constraints examined here are optimal, and indeed the optimal constraint would likely vary for different conditions and individuals. Undoubtedly, more research on different sets of kinematic constraints will shed light on how they can affect the spine motion during different activities.

## Conclusions

Taken together, our findings suggest that adding more spinal DOF up to 6DOF produces meaningful improvements in marker error and that kinematic constraints from 4 to 6DOF provide similar levels of kinematic smoothness that are better than unconstrained models. Moreover, all the constraint conditions examined were similarly valid in matching separately determined static segmental angles. Thus, adding more DOF (up to 9DOF) did not enhance the model's kinematic validity. Finally, on average, the joint ROMs produced the kinematic constraints from 4 to 6DOF were generally within physiologically reasonable ranges. These results indicate that the 5DOF model produces the best overall balance between the smoothness, realism of movement, and error of spine markers. The 6DOF model can provide a slight improvement in marker error during AR motions but at the expense of possibly less realistic LB motion patterns. It should be noted that most of the previous model validations focused on kinetic validations (e.g., Han et al., 2012; Bruno et al., 2015; Ignasiak et al., 2016), assessing whether a model can accurately predict musculoskeletal forces and muscle activations, by static optimization. These analyses require spinal kinematics to be specified as an input and are appropriately performed in models without kinematic constraints. However, our current work is a novel study that, for the first time, addresses the kinematic validity, whether a model precisely estimates the spine kinematics from inverse kinematics analysis through marker motion data. Our findings showed that this analysis is better performed in a model with kinematic constraints. Therefore, it is appropriate to utilize different versions of a model (with and without kinematic constraints) to evaluate kinematic and kinetic outcomes, respectively. In the future, additional research is warranted to understand the influence of kinematic constraints on the evaluation of spine motion during functional tasks and activities of daily living and in patients with spine disorders.

## Data Availability Statement

The raw data supporting the conclusions of this article will be made available by the authors, without undue reservation.

## Ethics Statement

The studies involving human participants were reviewed and approved by Institutional Review Board of Beth Israel Deaconess Medical Center, Boston, United States. The patients/participants provided their written informed consent to participate in this study.

## Author Contributions

DA, MB, and MA substantially contributed to the conception and design of the study. DA and SM contributed to the collection and processing of data and static pose analyses using marker clusters. AL processed marker data for dynamic trials. CZ and DA performed a literature review and analysis to specify the kinematic constraints used. KB, AL, and BA created subject-specific models, implemented kinematic constraints in the models, and performed inverse kinematics analyses. MA and DA performed statistical analyses. MA wrote the first draft of the manuscript. All authors critically revised the manuscript and approved the version to be published.

## Author Disclaimer

The content is solely the responsibility of the authors and does not necessarily represent the official views of Harvard Catalyst, Harvard University, and its affiliated academic healthcare centers, the National Institutes of Health, or the Department of Defense. The study sponsors had no role in the study design, data collection, analysis, manuscript preparation, or the decision to submit the manuscript for publication.

## Conflict of Interest

The authors declare that the research was conducted in the absence of any commercial or financial relationships that could be construed as a potential conflict of interest.

## Publisher's Note

All claims expressed in this article are solely those of the authors and do not necessarily represent those of their affiliated organizations, or those of the publisher, the editors and the reviewers. Any product that may be evaluated in this article, or claim that may be made by its manufacturer, is not guaranteed or endorsed by the publisher.
