# Supplementary material for: The Influence of Kinematic Constraints on Model Performance During Inverse Kinematics Analysis of the Thoracolumbar Spine
Source: Front Bioeng Biotechnol. 2021 Jul 29;9:688041. doi: 10.3389/fbioe.2021.688041 (PMC8358679; doi:10.3389/fbioe.2021.688041)
Supplement: Supplementary file 1 [file Data_Sheet_1.pdf]

Appendix A

Table 1

Percentage of intervertebral joint motions to overall thoracic spine motion in different studies used to create kinematic constraints in flexion-extension, lateral bending and axial rotation tasks.

Joint Level	Flexion-Extension				Lateral Bending		Axial Rotation	
	Mannion et al. (2004)	Morita et al. (2014)	White & Panjabi (1978)	Willems et al. (1996)	Fujimori et al. (2014)	White & Panjabi (1978)	Fujimori et al. (2012)	White & Panjabi (1978)
T12/L1	17.49%	13.25%	15.79%	9.63%	12.36%	10.26%	2.34%	2.70%
T11/T12	21.95%	11.99%	15.79%	9.63%	12.92%	11.54%	6.07%	2.70%
T10/T11	20.79%	11.36%	11.84%	9.63%	10.11%	8.97%	12.15%	2.70%
T9/T10	13.37%	10.41%	7.89%	9.63%	8.99%	7.69%	12.62%	5.41%
T8/T9	3.96%	8.52%	7.89%	8.26%	7.30%	7.69%	11.68%	9.46%
T7/T8	-1.16%	5.99%	7.89%	8.26%	9.55%	7.69%	10.75%	10.81%
T6/T7	2.15%	6.62%	6.58%	8.26%	6.18%	7.69%	8.88%	10.81%
T5/T6	7.26%	4.73%	5.26%	8.26%	4.49%	7.69%	8.41%	10.81%
T4/T5	4.13%	2.84%	5.26%	7.11%	5.06%	7.69%	7.48%	10.81%
T3/T4	0.33%	3.79%	5.26%	7.11%	7.87%	7.69%	6.54%	10.81%
T2/T3	10.23%	8.20%	5.26%	7.11%	7.30%	7.69%	7.48%	10.81%
T1/T2	-0.50%	12.30%	5.26%	7.11%	7.87%	7.69%	5.61%	12.16%

Table 2

Percentage of intervertebral joint motions to overall lumbar spine motion in different studies used to create kinematic constraints in flexion-extension task. Note that in study conducted by Cheng et al. (2016), two separate measurement techniques were used: Standard bending radiographs (SBR) and Vertebral Motion Analysis (VMA).

Joint Level	Flexion-Extension						
	Cheng et al. (2016)		Mannion et al. (2004)	Panjabi et al. (1994)	Rozumalski et al. (2008)	White & Panjabi (1978)	Wong et al. (2006)
	SBR	VMA					
L5/S1	16.56%	22.15%	17.98%	25.49%	17.91%	25.64%	10.51%
L4/L5	24.34%	17.85%	16.65%	22.28%	21.08%	21.79%	14.72%
L3/L4	20.65%	19.78%	28.42%	17.47%	22.14%	19.23%	20.35%
L2/L3	22.90%	21.72%	22.04%	18.72%	20.89%	17.95%	24.62%
L1/L2	15.54%	18.49%	14.91%	16.04%	17.97%	15.38%	29.79%

Table 3

Percentage of intervertebral joint motions to overall lumbar spine motion in different studies used to create kinematic constraints in lateral bending task. Note that in study conducted by Cheng et al. (2016), two separate measurement techniques were used: Standard bending radiographs (SBR) and Vertebral Motion Analysis (VMA).

Joint Level	Lateral Bending					
	Cheng et al. (2016)		Panjabi et al. (1994)	Pearcy & Tibrewal (1984)	Rozumalski et al. (2008)	White & Panjabi (1978)
	SBR	VMA				
L5/S1	5.35%	7.85%	17.96%	7.50%	13.55%	10.34%
L4/L5	20.28%	21.77%	19.56%	15.00%	18.11%	20.69%
L3/L4	28.73%	25.57%	20.96%	25.00%	24.53%	27.59%
L2/L3	27.32%	24.30%	23.95%	27.50%	25.01%	20.69%
L1/L2	18.31%	20.51%	17.56%	25.00%	18.79%	20.69%

Table 4

Percentage of intervertebral joint motions to overall lumbar spine motion in different studies used to create kinematic constraints in axial rotation task.

Joint Level	Axial Rotation					
	**Fujii et al. (2007)**	**Panjabi et al. (1994)**	**Pearcy & Tibrewal (1984)**	**Rozumalski et al. (2008)**	**Shin et al. (2013)**	**White & Panjabi (1978)**
L5/S1	20.78%	11.93%	16.67%	19.19%	20.92%	38.46%
L4/L5	22.08%	18.18%	25.00%	20.07%	20.92%	15.38%
L3/L4	22.08%	25.00%	25.00%	18.37%	14.38%	15.38%
L2/L3	18.18%	25.00%	16.67%	20.97%	21.90%	15.38%
L1/L2	16.88%	19.89%	16.67%	21.40%	21.90%	15.38%
