# Supplementary material for: The Influence of Kinematic Constraints on Model Performance During Inverse Kinematics Analysis of the Thoracolumbar Spine
Source: Front Bioeng Biotechnol. 2021 Jul 29;9:688041. doi: 10.3389/fbioe.2021.688041 (PMC8358679; doi:10.3389/fbioe.2021.688041)

# Appendix B

## Table 1

Ratios of individual-level intervertebral motions to overall spine motion used to develop the kinematic constraints (i.e., 3-9DOF) in three tasks (i.e., flexion-extension, lateral bending and axial rotation). Three colors (blue, green, and orange) were used in this table to demonstrate the number of DOF in each rotational direction. The columns with one, two and three colors demonstrated one, two and three DOFs in each rotational direction, respectively.

| **Joint Level** | 3DOF (1 FE, 1 LB, 1 AR) | | | 4DOF (2 FE, 1 LB, 1 AR) | | | 5DOF (2 FE, 2 LB, 1 AR) | | | 6DOF (2 FE, 2 LB, 2 AR) | | | 7DOF (2 FE, 2 LB, 3 AR) | | | 8DOF (2 FE, 3 LB, 3 AR) | | | 9DOF (3 FE, 3 LB, 3 AR) | | |
|---|---|---|---|---|---|---|---|---|---|---|---|---|---|---|---|---|---|---|---|---|---|
| | **Flex-Ext** | **Lat Bend** | **Axial Rot** | **Flex-Ext** | **Lat Bend** | **Axial Rot** | **Flex-Ext** | **Lat Bend** | **Axial Rot** | **Flex-Ext** | **Lat Bend** | **Axial Rot** | **Flex-Ext** | **Lat Bend** | **Axial Rot** | **Flex-Ext** | **Lat Bend** | **Axial Rot** | **Flex-Ext** | **Lat Bend** | **Axial Rot** |
| **L5/S1** | 0.13 | 0.04 | 0.04 | 0.15 | 0.04 | 0.04 | 0.15 | 0.09 | 0.04 | 0.15 | 0.09 | 0.20 | 0.15 | 0.09 | 0.20 | 0.15 | 0.09 | 0.20 | 0.20 | 0.09 | 0.20 |
| **L4/L5** | 0.15 | 0.08 | 0.04 | 0.18 | 0.08 | 0.04 | 0.18 | 0.20 | 0.04 | 0.18 | 0.20 | 0.21 | 0.18 | 0.20 | 0.21 | 0.18 | 0.20 | 0.21 | 0.21 | 0.20 | 0.21 |
| **L3/L4** | 0.15 | 0.10 | 0.04 | 0.17 | 0.10 | 0.04 | 0.17 | 0.26 | 0.04 | 0.17 | 0.26 | 0.20 | 0.17 | 0.26 | 0.20 | 0.17 | 0.26 | 0.20 | 0.20 | 0.26 | 0.20 |
| **L2/L3** | 0.16 | 0.10 | 0.04 | 0.19 | 0.10 | 0.04 | 0.19 | 0.25 | 0.04 | 0.19 | 0.25 | 0.20 | 0.19 | 0.25 | 0.20 | 0.19 | 0.25 | 0.20 | 0.20 | 0.25 | 0.20 |
| **L1/L2** | 0.12 | 0.08 | 0.04 | 0.14 | 0.08 | 0.04 | 0.14 | 0.20 | 0.04 | 0.14 | 0.20 | 0.19 | 0.14 | 0.20 | 0.19 | 0.14 | 0.20 | 0.19 | 0.19 | 0.20 | 0.19 |
| **T12/L1** | 0.04 | 0.07 | 0.02 | 0.04 | 0.07 | 0.02 | 0.04 | 0.11 | 0.02 | 0.04 | 0.11 | 0.03 | 0.04 | 0.11 | 0.11 | 0.04 | 0.16 | 0.11 | 0.25 | 0.16 | 0.11 |
| **T11/T12** | 0.04 | 0.07 | 0.04 | 0.05 | 0.07 | 0.04 | 0.05 | 0.12 | 0.04 | 0.05 | 0.12 | 0.04 | 0.05 | 0.12 | 0.19 | 0.05 | 0.17 | 0.19 | 0.27 | 0.17 | 0.19 |
| **T10/T11** | 0.04 | 0.06 | 0.06 | 0.05 | 0.06 | 0.06 | 0.05 | 0.10 | 0.06 | 0.05 | 0.10 | 0.07 | 0.05 | 0.10 | 0.32 | 0.05 | 0.14 | 0.32 | 0.27 | 0.14 | 0.32 |
| **T9/T10** | 0.03 | 0.05 | 0.07 | 0.03 | 0.05 | 0.07 | 0.03 | 0.08 | 0.07 | 0.03 | 0.08 | 0.09 | 0.03 | 0.08 | 0.39 | 0.03 | 0.12 | 0.39 | 0.21 | 0.12 | 0.39 |
| **T8/T9** | 0.02 | 0.04 | 0.09 | 0.13 | 0.04 | 0.09 | 0.13 | 0.07 | 0.09 | 0.13 | 0.07 | 0.11 | 0.13 | 0.07 | 0.14 | 0.13 | 0.11 | 0.14 | 0.13 | 0.11 | 0.14 |
| **T7/T8** | 0.02 | 0.05 | 0.09 | 0.12 | 0.05 | 0.09 | 0.12 | 0.09 | 0.09 | 0.12 | 0.09 | 0.11 | 0.12 | 0.09 | 0.14 | 0.12 | 0.12 | 0.14 | 0.12 | 0.12 | 0.14 |
| **T6/T7** | 0.01 | 0.04 | 0.08 | 0.10 | 0.04 | 0.08 | 0.10 | 0.07 | 0.08 | 0.10 | 0.07 | 0.10 | 0.10 | 0.07 | 0.13 | 0.10 | 0.10 | 0.13 | 0.10 | 0.10 | 0.13 |
| **T5/T6** | 0.02 | 0.04 | 0.08 | 0.11 | 0.04 | 0.08 | 0.11 | 0.06 | 0.08 | 0.11 | 0.06 | 0.10 | 0.11 | 0.06 | 0.13 | 0.11 | 0.09 | 0.13 | 0.11 | 0.09 | 0.13 |
| **T4/T5** | 0.01 | 0.04 | 0.07 | 0.06 | 0.04 | 0.07 | 0.06 | 0.06 | 0.07 | 0.06 | 0.06 | 0.09 | 0.06 | 0.06 | 0.12 | 0.06 | 0.22 | 0.12 | 0.06 | 0.22 | 0.12 |
| **T3/T4** | 0.02 | 0.05 | 0.07 | 0.11 | 0.05 | 0.07 | 0.11 | 0.08 | 0.07 | 0.11 | 0.08 | 0.09 | 0.11 | 0.08 | 0.11 | 0.11 | 0.26 | 0.11 | 0.11 | 0.26 | 0.11 |
| **T2/T3** | 0.02 | 0.04 | 0.07 | 0.17 | 0.04 | 0.07 | 0.17 | 0.07 | 0.07 | 0.17 | 0.07 | 0.09 | 0.17 | 0.07 | 0.12 | 0.17 | 0.25 | 0.12 | 0.17 | 0.25 | 0.12 |
| **T1/T2** | 0.03 | 0.05 | 0.07 | 0.20 | 0.05 | 0.07 | 0.20 | 0.08 | 0.07 | 0.20 | 0.08 | 0.09 | 0.20 | 0.08 | 0.12 | 0.20 | 0.26 | 0.12 | 0.20 | 0.26 | 0.12 |

## Figure 1

Comparison of averaged angular motions of spine segments (i.e., T1-T5, T5-T9, T9-L1, L1-S1) across different kinematic constraints (3-9DOF and baseline or no constraint (i.e., 51DOF)) in primary direction (FE) of flexion-extension task. The time was normalized to the scale of 0 to 100% of the task.

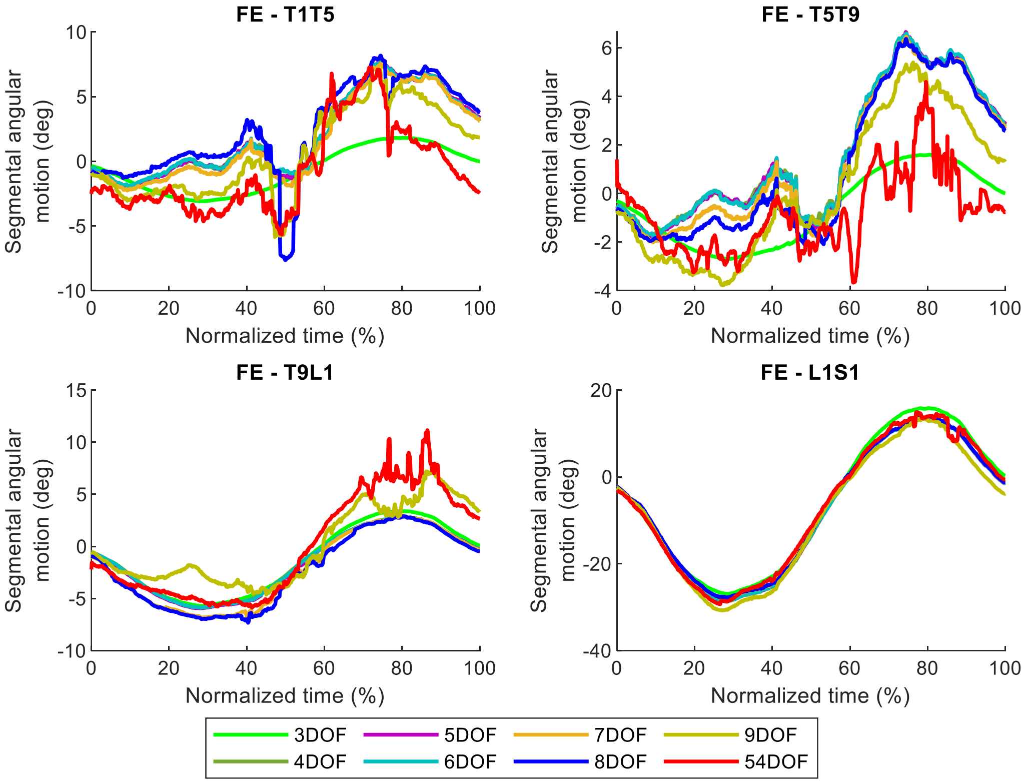

**Figure 2**

Comparison of averaged angular motions of spine segments (i.e., T1-T5, T5-T9, T9-L1, L1-S1) across different kinematic constraints (3-9DOF and baseline or no constraint (i.e., 51DOF)) in primary direction (LB) of lateral bending task. The time was normalized to the scale of 0 to 100% of the task.

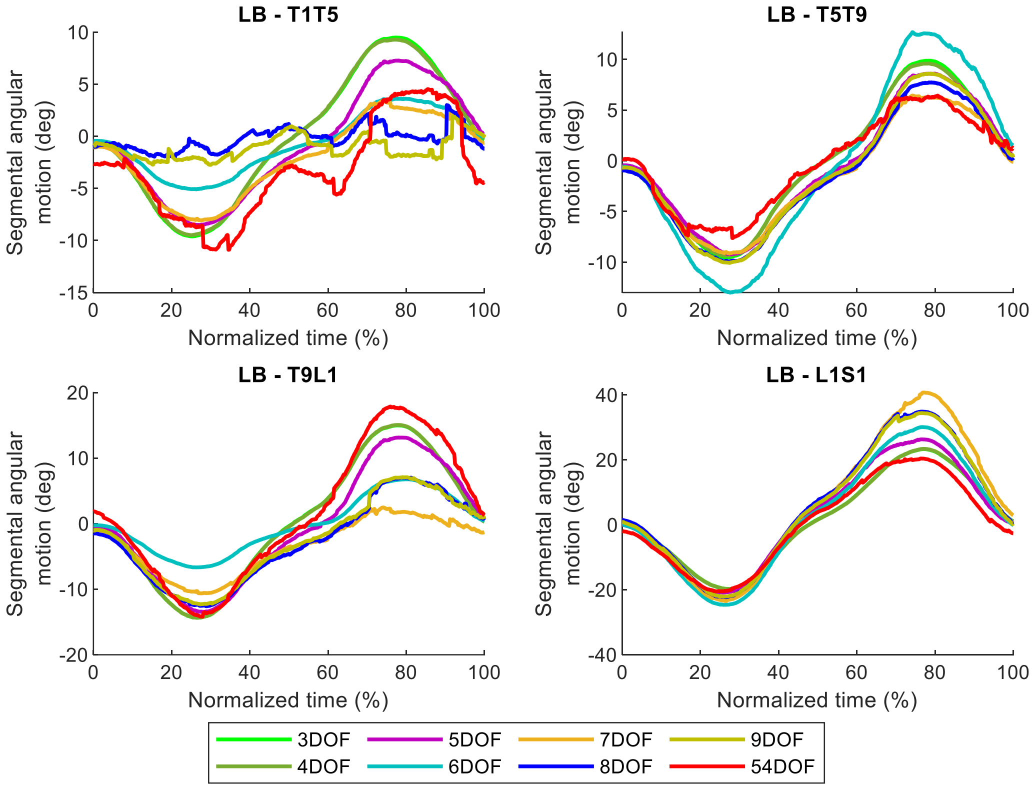

**Figure 3**

Comparison of averaged angular motions of spine segments (i.e., T1-T5, T5-T9, T9-L1, L1-S1) across different kinematic constraints (3-9DOF and baseline or no constraint (i.e., 51DOF)) in primary direction (AR) of axial rotation task. The time was normalized to the scale of 0 to 100% of the task.

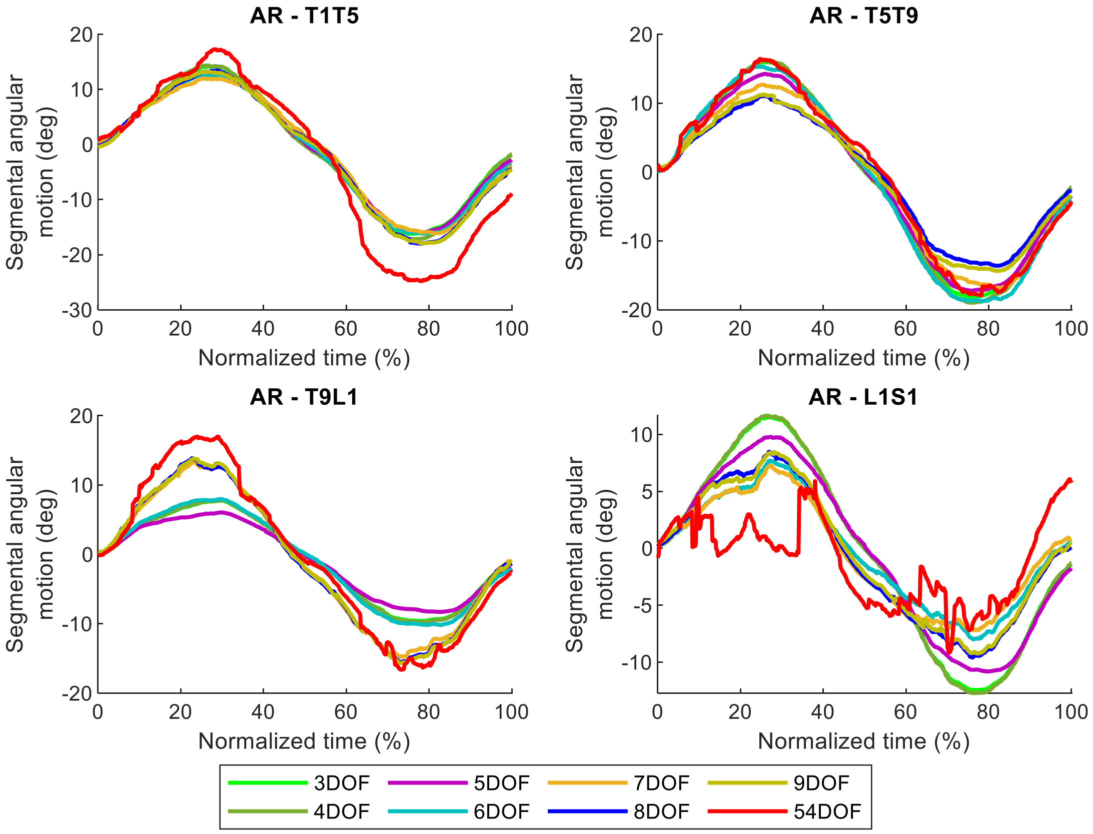

Supplement: Supplementary file 2 [file Data_Sheet_2.pdf]
