# Supplementary material for: The Influence of Kinematic Constraints on Model Performance During Inverse Kinematics Analysis of the Thoracolumbar Spine
Source: Front Bioeng Biotechnol. 2021 Jul 29;9:688041. doi: 10.3389/fbioe.2021.688041 (PMC8358679; doi:10.3389/fbioe.2021.688041)
Supplement: Supplementary file 3 [file Data_Sheet_3.pdf]

Appendix C

Table 1

Range (represented in brackets as [min,max]) of maximum ROM of lumbar intervertebral joint angles (across four tasks: flexion, extension, lateral bending, axial rotation) for each combination of kinematic constraints (3-9DOF and no constraint (51DOF)) and direction of motion (FE, LB, AR).

Motion Direction	DOF	L1/L2	L2/L3	L3/L4	L4/L5	L5/S1
FE	3	[2.2,12.1]	[2.9,16.2]	[2.7,15.2]	[2.5,14.2]	[2.4,13.1]
	4	[2.2,13.5]	[3.0,18.4]	[2.7,16.4]	[2.8,17.4]	[2.4,14.5]
	5	[1.8,13.5]	[2.4,18.3]	[2.1,16.4]	[2.3,17.3]	[1.9,14.4]
	6	[1.7,13.5]	[2.4,18.3]	[2.1,16.4]	[2.2,17.3]	[1.9,14.5]
	7	[1.9,13.5]	[2.5,18.3]	[2.3,16.4]	[2.4,17.4]	[2.0,14.5]
	8	[1.9,13.5]	[2.6,18.4]	[2.3,16.4]	[2.5,17.4]	[2.1,14.5]
	9	[2.1,15.8]	[2.7,20.5]	[2.6,19.6]	[2.6,19.6]	[2.3,17.7]
	51	[17.8,45.6]	[6.3,17.0]	[9.1,32.1]	[9.4,28.6]	[21.2,69.6]
LB	3	[6.3,11.5]	[7.8,14.4]	[7.8,14.4]	[6.3,11.5]	[3.1,5.7]
	4	[6.3,11.5]	[7.8,14.4]	[7.8,14.4]	[6.3,11.5]	[3.1,5.8]
	5	[5.9,15.8]	[7.4,19.7]	[7.7,20.5]	[5.9,15.8]	[2.7,7.1]
	6	[7.5,19.3]	[9.4,24.1]	[9.7,25.1]	[7.5,19.3]	[3.4,8.7]
	7	[8.4,18.0]	[10.5,22.4]	[10.9,23.3]	[8.4,18.0]	[3.8,8.1]
	8	[7.8,17.4]	[9.8,21.8]	[10.2,22.7]	[7.8,17.4]	[3.5,7.8]
	9	[7.5,17.0]	[9.4,21.3]	[9.8,22.1]	[7.5,17.0]	[3.4,7.7]
	51	[4.4,45.5]	[4.0,25.1]	[3.8,27.2]	[6.9,24.5]	[7.0,37.0]
AR	3	[3.4,6.4]	[3.4,6.4]	[3.4,6.4]	[2.5,4.8]	[3.4,6.4]
	4	[3.4,6.4]	[3.4,6.4]	[3.4,6.4]	[2.5,4.8]	[3.4,6.4]
	5	[2.9,5.4]	[2.9,5.4]	[2.9,5.4]	[2.2,4.0]	[2.9,5.4]
	6	[1.8,7.2]	[1.8,7.6]	[1.8,7.6]	[1.9,8.0]	[1.8,7.6]
	7	[1.9,6.5]	[2.0,6.9]	[2.0,6.9]	[2.1,7.2]	[2.0,6.9]
	8	[2.6,6.8]	[2.7,7.1]	[2.7,7.1]	[2.9,7.5]	[2.7,7.1]
	9	[2.6,6.9]	[2.7,7.2]	[2.7,7.2]	[2.9,7.6]	[2.7,7.2]
	51	[2.4,27.0]	[2.6,17.3]	[3.0,11.2]	[2.7,16.0]	[4.4,36.5]

Table 2

Range (represented in brackets as [min,max]) of maximum ROM of thoracic intervertebral joint angles (across four tasks: flexion, extension, lateral bending, axial rotation) for each combination of kinematic constraints (3-9DOF and no constraint (51DOF)) and direction of motion (FE, LB, AR).

Motion Direction	DOF	T1/T2	T2/T3	T3/T4	T4/T5	T5/T6	T6/T7	T7/T8	T8/T9	T9/T10	T10/T11	T11/T12	T12/L1
FE	3	[.5,3.0]	[.4,2.0]	[.4,2.0]	[.2,1.0]	[.4,2.0]	[.2,1.0]	[.4,2.0]	[.4,2.0]	[.5,3.0]	[.7,4.0]	[.7,4.0]	[.7,4.0]
	4	[2.7,9.5]	[2.3,8.1]	[1.5,5.2]	[.8,2.9]	[1.5,5.2]	[1.4,4.8]	[1.6,5.7]	[1.8,6.2]	[.5,2.9]	[.8,4.8]	[.8,4.8]	[.6,3.9]
	5	[3.1,9.4]	[2.6,8.0]	[1.7,5.1]	[.9,2.8]	[1.7,5.1]	[1.5,4.7]	[1.9,5.6]	[2.0,6.1]	[.4,2.9]	[.6,4.8]	[.6,4.8]	[.5,3.9]
	6	[2.5,9.6]	[2.1,8.1]	[1.4,5.3]	[.8,2.9]	[1.4,5.3]	[1.3,4.8]	[1.5,5.7]	[1.6,6.2]	[.4,2.9]	[.6,4.8]	[.6,4.8]	[.5,3.9]
	7	[2.5,9.2]	[2.2,7.8]	[1.4,5.1]	[.8,2.8]	[1.4,5.1]	[1.3,4.6]	[1.5,5.5]	[1.7,6.0]	[.4,2.9]	[.7,4.8]	[.7,4.8]	[.5,3.9]
	8	[2.3,7.8]	[2.0,6.6]	[1.3,4.3]	[.7,2.3]	[1.3,4.3]	[1.1,3.9]	[1.4,4.7]	[1.5,5.1]	[.4,2.9]	[.7,4.8]	[.7,4.8]	[.5,3.9]
	9	[2.3,7.4]	[2.0,6.3]	[1.3,4.1]	[.7,2.2]	[1.3,4.1]	[1.2,3.7]	[1.4,4.5]	[1.5,4.8]	[1.7,5.8]	[2.2,7.5]	[2.2,7.5]	[2.0,6.9]
	51	[15.0,34.7]	[2.5,20.9]	[17.7,39.6]	[2.4,26.4]	[14.3,40.1]	[1.2,10.1]	[15.3,29.2]	[1.2,17.3]	[13.9,31.9]	[3.9,13.4]	[13.3,40.4]	[1.8,38.6]
LB	3	[3.9,7.2]	[3.1,5.7]	[3.9,7.2]	[3.1,5.7]	[3.1,5.7]	[3.1,5.7]	[3.9,7.2]	[3.1,5.7]	[3.9,7.2]	[4.7,8.6]	[5.5,10.1]	[5.5,10.1]
	4	[3.9,7.2]	[3.1,5.8]	[3.9,7.2]	[3.1,5.8]	[3.1,5.8]	[3.1,5.8]	[3.9,7.2]	[3.1,5.8]	[3.9,7.2]	[4.7,8.6]	[5.5,10.1]	[5.5,10.1]
	5	[3.1,7.4]	[2.7,6.5]	[3.1,7.4]	[2.3,5.5]	[2.3,5.5]	[2.7,6.5]	[3.9,9.2]	[2.7,6.5]	[3.1,7.4]	[3.9,9.2]	[4.7,11.1]	[4.3,10.1]
	6	[1.4,2.9]	[1.3,2.5]	[1.4,2.9]	[1.1,2.2]	[1.1,2.2]	[1.3,2.5]	[1.8,3.6]	[1.3,2.5]	[1.4,2.9]	[1.8,3.6]	[2.1,4.4]	[2.0,4.0]
	7	[2.5,5.6]	[2.2,4.9]	[2.5,5.6]	[1.9,4.2]	[1.9,4.2]	[2.2,4.9]	[3.1,7.0]	[2.2,4.9]	[2.5,5.6]	[3.1,7.0]	[3.7,8.3]	[3.4,7.7]
	8	[4.9,32.1]	[4.7,30.9]	[5.1,33.4]	[4.2,27.2]	[2.2,10.8]	[2.5,12.0]	[2.7,13.2]	[2.7,13.2]	[3.0,14.4]	[3.5,16.8]	[4.2,20.4]	[4.0,19.2]
	9	[3.8,19.7]	[3.6,18.9]	[3.9,20.5]	[3.2,16.7]	[2.5,7.4]	[2.8,8.3]	[3.0,9.1]	[3.0,9.1]	[3.3,9.9]	[3.9,11.6]	[4.7,14.0]	[4.4,13.2]
	51	[11.2,60.7]	[3.5,17.0]	[13.1,66.9]	[4.1,58.1]	[6.5,42.2]	[3.5,17.2]	[8.4,53.9]	[4.8,50.3]	[4.9,49.4]	[4.2,18.4]	[5.1,52.8]	[5.0,71.1]
AR	3	[5.9,11.2]	[5.9,11.2]	[5.9,11.2]	[5.9,11.2]	[6.8,12.8]	[6.8,12.8]	[7.6,14.4]	[7.6,14.4]	[5.9,11.2]	[5.1,9.6]	[3.4,6.4]	[1.7,3.2]
	4	[5.9,11.3]	[5.9,11.3]	[5.9,11.3]	[5.9,11.3]	[6.8,12.9]	[6.8,12.9]	[7.6,14.5]	[7.6,14.5]	[5.9,11.3]	[5.1,9.7]	[3.4,6.4]	[1.7,3.2]
	5	[5.1,9.4]	[5.1,9.4]	[5.1,9.4]	[5.1,9.4]	[5.9,10.7]	[5.9,10.7]	[6.6,12.1]	[6.6,12.1]	[5.1,9.4]	[4.4,8.0]	[2.9,5.4]	[1.5,2.7]
	6	[5.1,9.6]	[5.1,9.6]	[5.1,9.6]	[5.1,9.6]	[5.7,10.7]	[5.7,10.7]	[5.7,10.7]	[6.3,11.7]	[5.1,9.6]	[4.0,7.5]	[2.3,4.3]	[1.7,3.2]
	7	[4.8,9.7]	[4.8,9.7]	[4.4,8.9]	[4.8,9.7]	[5.2,10.5]	[5.2,10.5]	[5.2,10.5]	[5.6,11.3]	[9.4,18.1]	[7.7,14.9]	[4.3,8.4]	[2.6,5.1]
	8	[4.5,11.0]	[4.5,11.0]	[4.1,10.0]	[4.5,11.0]	[4.8,11.9]	[4.8,11.9]	[4.8,11.9]	[5.2,12.8]	[9.4,17.5]	[7.7,14.3]	[4.3,8.1]	[2.7,4.9]
	9	[4.3,7.9]	[4.3,7.9]	[3.9,7.3]	[4.3,7.9]	[4.7,8.6]	[4.7,8.6]	[4.7,8.6]	[5.0,9.3]	[9.6,17.5]	[7.9,14.4]	[4.4,8.1]	[2.7,4.9]
	51	[8.5,31.1]	[6.9,24.3]	[6.8,32.9]	[4.2,30.7]	[4.8,20.4]	[4.8,18.2]	[4.8,18.7]	[5.7,19.0]	[3.8,24.2]	[4.4,16.1]	[4.5,22.1]	[5.2,44.9]
